# Contra Procrustes’ medicine: ars medica in the era of tags and labels

**DOI:** 10.1186/s44158-023-00108-4

**Published:** 2023-07-31

**Authors:** Rocco Pace, Giacomo Bellani

**Affiliations:** 1 Department of Anesthesia and Intensive Care, Santa Chiara Hospital, APSS Trento, Trento, Italy; 2 Centre for Medical Sciences CISMed, University of Trento, Trento, Italy

**Keywords:** Evidence-based medicine, Medical education, Protocols, Outcomes

Medicine can be properly considered a “Science” only until we consider Medicine in theory, medicine as a concept.

But if we consider medicine as the actions we take on the sick patient, then Medicine tends to lose some of the properties of science becoming more of a “Medical Art”.

Indeed, all scientific processes share two fundamental features: measurability and reproducibility. Medicine is certainly characterized by measurability; nevertheless, it is, frequently, weak in reproducibility, owing to the tremendous interindividual variability: every human being is unique and even identical twins are different.

The object of interest of Medicine, the patient, is a unique human being: unique as an organism and in terms of history, experience, and relationships (familial and social); unique as to what concerns their clinical history (e.g., underlying medical conditions).

It has become more and more common for modern doctors to simplistically classify, label, and treat their patients according to their specific condition. As a result, these patients will be assimilated into structured paths (protocols) and will keep following those paths. This reminds us of Procrustes.

In Greek mythology, Procrustes (Fig. 1A) was a very famous brigand, either legend or reality. He was very active on the Sacred Way, the road between Athens and Eleusis. He used to hide along that road and assault unfortunate travelers, robbing them of all their possessions and finally torturing them.

**Fig. 1:**
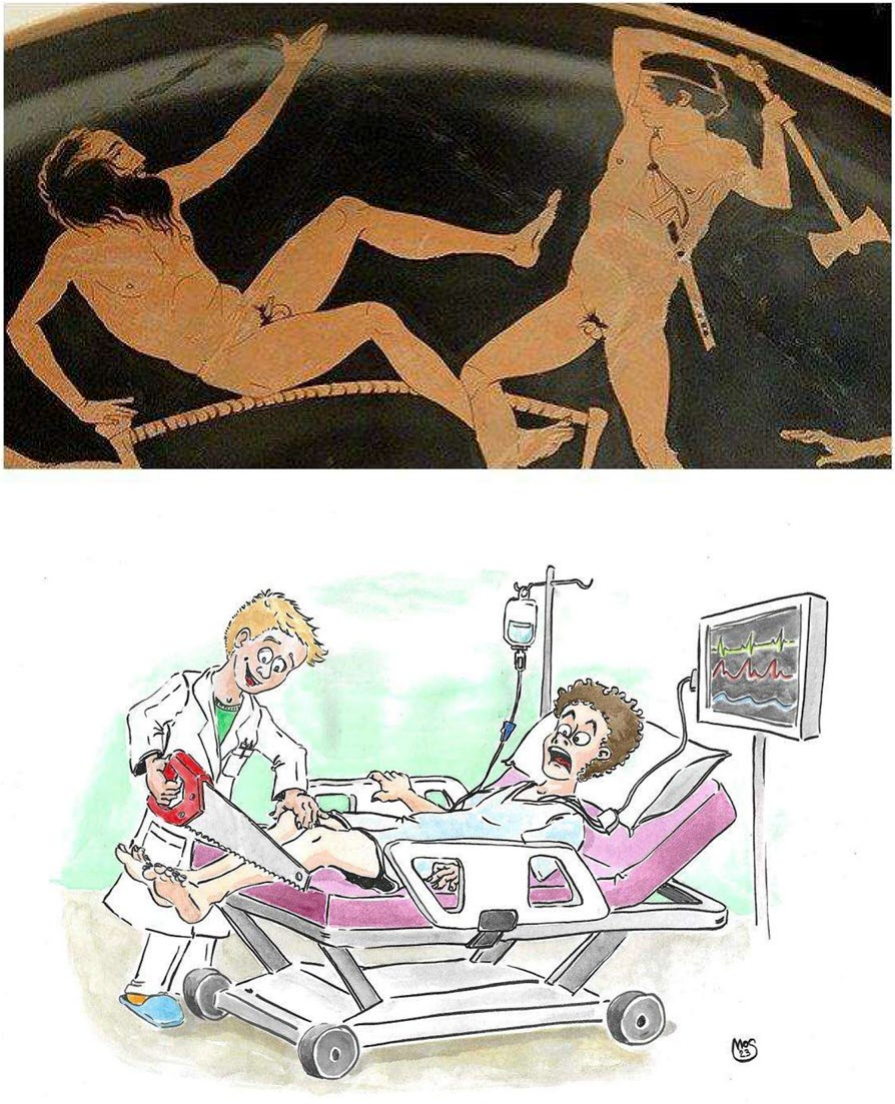
Procustes’ bed. Original version, showing Procustes being killed on his own bed by Theseus (**A** The Trustees of the British Museum. Shared under a Creative Commons Attribution-NonCommercial-ShareAlike 4.0 International (CC BY-NC-SA 4.0) licence) and modern (**B**) version

According to some authors, Procruste’s torture bed was wooden; according to others, it was made of rock. The bed had standard width and length and Procrustes used to make his victims lie on it forcing them to fit the bed by cutting off the parts that hung off the ends or, on the opposite, by stretching those people who were too short.

This “Procrustean sin” unfortunately afflicts several younger doctors in these days (Fig. 1B), making its way thanks to their uncertainties and leading them to strictly embrace defensive medicine, one of the evils of today’s medicine.

A spasmodical look for protocols [1, 2] on which to rely and stick to (sometimes complain about the lack of protocol for every possible condition), and labeling patients according to one specific condition (e.g., pulmonary embolism, and septic shock), leads to forcing their patients to “fit the bed,” cutting off or stretching where necessary. Forgetting the human being, forgetting that Medicine is, and always will be, the Art of doing the best possible to help our patients with the best resources available when and where we are. It should also be kept in mind to avoid a “Procustean approach” in defining what is “the best for our patients”: while several studies look at “survival” as a primary endpoint, survival might not be always the most valued outcome for a patient.

Mature doctors must of course know medicine as a science and keep themselves updated with literature, but they should never lose their curiosity [3] and humbly practice Medicine as an Art.

## Data Availability

Not applicable.
